# Open-Path Cavity Ring-Down Spectroscopy for Simultaneous Detection of Hydrogen Chloride and Particles in Cleanroom Environment

**DOI:** 10.3390/s24175611

**Published:** 2024-08-29

**Authors:** Muhammad Bilal Khan, Christian L’Orange, Cheongha Lim, Deokhyeon Kwon, Azer P. Yalin

**Affiliations:** 1 Department of Mechanical Engineering, Colorado State University, Fort Collins, CO 80525, USA; bilalk@rams.colostate.edu (M.B.K.); christian.lorange@colostate.edu (C.L.); 2 Pyeongtaek Infra Analysis Group, Infra Analysis Team, Samsung Electronics, Pyeongtaek-si 17786, Republic of Korea; cheong.lim@samsung.com (C.L.); deo.kwon@samsung.com (D.K.)

**Keywords:** airborne molecular contaminant, AMC, hydrogen chloride, HCl, particles, cavity ring-down spectroscopy, CRDS, open path, cleanroom, contamination

## Abstract

The present study addresses advanced monitoring techniques for particles and airborne molecular contaminants (AMCs) in cleanroom environments, which are crucial for ensuring the integrity of semiconductor manufacturing processes. We focus on quantifying particle levels and a representative AMC, hydrogen chloride (HCl), having known detrimental effects on equipment longevity, product yield, and human health. We have developed a compact laser sensor based on open-path cavity ring-down spectroscopy (CRDS) using a 1742 nm near-infrared diode laser source. The sensor enables the high-sensitivity detection of HCl through absorption by the 2-0 vibrational band with an Allan deviation of 0.15 parts per billion (ppb) over 15 min. For quantifying particle number concentrations, we examine various detection methods based on statistical analyses of Mie scattering-induced ring-down time fluctuations. We find that the ring-down distributions’ 3rd and 4th standard moments allow particle detection at densities as low as ~10^5^ m^−3^ (diameter > 1 μm). These findings provide a basis for the future development of compact cleanroom monitoring instrumentation for wafer-level monitoring for both AMC and particles, including mobile platforms.

## 1. Introduction

The semiconductor industry serves as the backbone of the multi-trillion-dollar electronics sector. Integrated circuits on semiconductors have consistently decreased in feature size to produce more efficient and faster chips. Given the tight production tolerances and limited room for error, suitable advanced manufacturing can only be completed in stringently controlled clean environments. Cleanroom monitoring refers to the continuous measurement and analysis of this controlled environment, which is commonly used in sectors such as pharmaceuticals, biotechnology, electronics, and aerospace [1,2].

Particulate matter (PM), such as dust, dirt, and other contaminants, can jeopardize the production of integrated circuits and semiconductor devices. Even slight variations in particulate contamination or gas concentrations can lead to device failures, machine downtimes, and significant yield losses. The International Organization for Standardization (ISO) maintains classifications on allowable concentration of particles in cleanrooms [3]. A main focus of the present work is the semiconductor manufacturing industry for which reducing airborne molecular contamination (AMC), i.e., certain gas-phase molecules, are critically important to reducing yield loss in the manufacture of integrated circuits. Indeed, several international technology roadmaps have highlighted the importance of AMC detection in manufacturing environments at part-per-billion (ppb) levels [4,5,6]. By employing robust PM and gas-phase measurement techniques [5,7,8,9], semiconductor manufacturers can proactively identify potential sources of contamination or process deviations at an early stage, optimize maintenance to ensure equipment functionality, minimize downtime, and reduce the risk of defects [10]. Measuring AMC and particle concentrations can also support compliance with environmental and workplace safety regulations.

**Figure 1:**
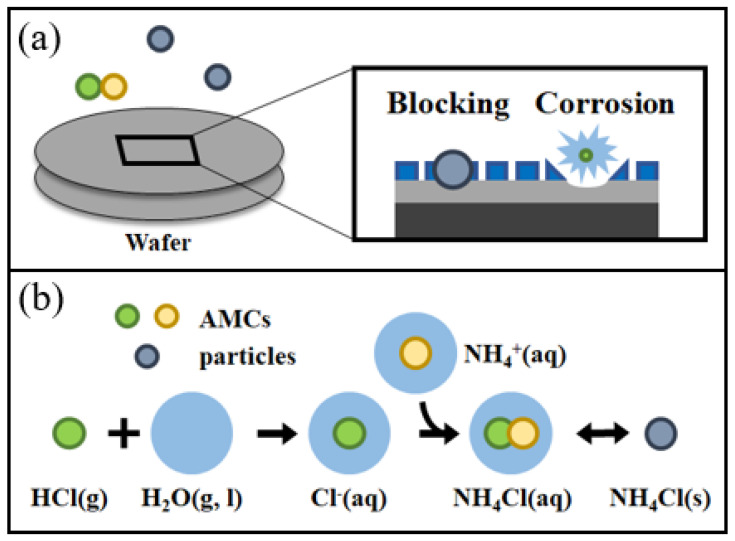
(**a**) Example mechanism of yield loss by particle and AMC on the wafer surface, and (**b**) reaction between AMCs for particle formation.

Depending on the specifics of the cleanroom, various airborne molecular contaminants (AMCs) can be significant. Two notable examples of these are the acidic gaseous molecules hydrogen fluoride (HF) and hydrogen chloride (HCl). Many manufacturing processes involve the use of halogen gas mixtures such as HCl. One critical issue in semiconductor manufacturing processes is halogen gas adsorption on substrates during plasma etching or deposition processes [11]. Failure to adequately purge these gases from the process chamber can result in a significant presence of adsorbed chlorine-based species on the film surface. Their presence on the wafer can cause patterning defects such as the localized blockage of subsequent plasma-etching processes (Figure 1a). The precise control over gas flow rates and composition ensures consistent and reliable outcomes, enhances production efficiency, and reduces the occurrence of defects. HCl is also known to impact device fabrication yield through copper (Cu) electrode corrosion and due to PM formation with ammonia molecules (leading to NH_4_Cl(s)), as shown in Figure 1b, potentially leading to the disconnection of circuits by blocking lines [12,13]. Furthermore, Pelissier et al. contaminated 200 nm Cu wafers with gaseous HCl and found that the presence of HCl results in the formation of (solid-phase) copper chlorohydroxide, which significantly restricts the functionality of the wafers [14]. Indeed, there are multiple examples of the relevance of gas-to-particle conversion, deriving from the presence of AMCs and often mediated by moisture, which can influence semiconductor manufacture (e.g., [15,16]). Another example of an AMC is gaseous ammonia (NH_3_), which affects the hygiene of optical surfaces of photolithography instrumentation and product (wafer) surfaces via particulate matter formation with anionic counter partners (e.g., SO_4_^2-^) and surface settlements [7,8].

The current industry standard for cleanroom PM monitoring is the use of optical counters that utilize particles’ light-scattering properties [17]. However, optical PM sensors can struggle at low PM levels where there are very few particles for the light to interact with. In these cases, ISO protocols allow for sequential sampling, but sampling and statistical limitations remain a challenge at low ISO levels [3]. For AMC monitoring in semiconductor manufacturing, the industry standard is a multifaceted approach using a suite of instruments based on techniques such as gas chromatography, mass spectrometry, Fourier transform infrared spectroscopy, ion mobility spectrometry techniques, or photoionization detection [18,19]. Depending on the exact application, specific methods (instruments) are chosen based on their complementary strengths and practical requirements such as size and cost.

**Figure 2:**
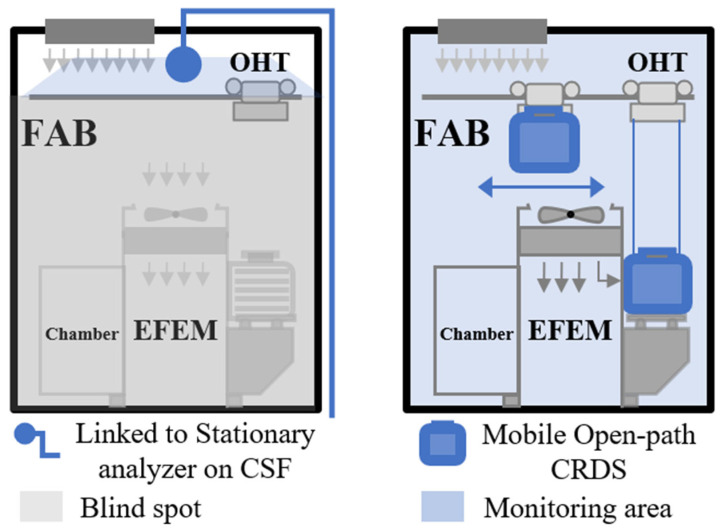
**Left**: Conventional monitoring system based on stationary analyzer and with limitation of blind spots. **Right**: Concept for mobile monitoring system using open-path CRDS allowing detection without blind spots. FAB: semiconductor fabrication area; EFEM: equipment front end module; OHT: overhead hoist transportation.

The present contribution develops a portable instrument (80 cm × 15 cm × 20 cm) for simultaneous particle and AMC detection which can be used as a part of future advanced mobile monitoring systems (Figure 2). The concept is to have an instrument set on a gantry system that can provide large-area coverage of different locations by physically moving the instrument into the region of interest, including the inside of manufacturing instrumentation. Such a system would be in contrast to stationary multi-port (manifold) sampling systems, which are not suitable for wafer-level monitoring due to characteristics including relatively slow time response, large volume, and high power consumption.

As described herein, the compact instrument is based on a novel implementation of open-path cavity ring-down spectroscopy (CRDS), which is an ultra-sensitive optical absorption diagnostic that can detect both PMs and AMCs. Cavity ring-down instruments can be made relatively compact because the long optical path allows for precise measurements within a small physical footprint, although cavity lengths of at least ~10 cm are typically required. The sensing time response, typically characterized with T_10–90_, meaning the time needed for the sensor to respond to a step-change in concentration (due to the gap between 10% and 90% of the recorded change), is a key sensor characteristic. For mobile sensing especially, a high (short) time response is critical for accurately locating emission sources. An advantage of the open-path CRDS that we apply, i.e., where the gas within the path of the optical head is directly sensed, is the possibility of near instantaneous time response, whereas conventional closed-path systems (with pump and inlet lines) tend to have poorer time responses due to the transit time as well as the possibility of (“sticky-gas“) adsorption effects on the inlet line or cell wall materials.

The layout of the remainder of the paper is as follows. Section 2 presents an overview of CRDS principles including the Beer–Lambert law and the measurement of absorption and scattering. Section 3 presents the experimental methods including the testbed chamber setup for controlled HCl and particle levels and the configuration of the open-path CRDS instrument. Section 4 provides results and discussion. Finally, Section 5 provides conclusions of key findings along with limitations and future research directions.

## 2. Overview of Cavity Ring-Down Spectroscopy (CRDS)

**Figure 3:**
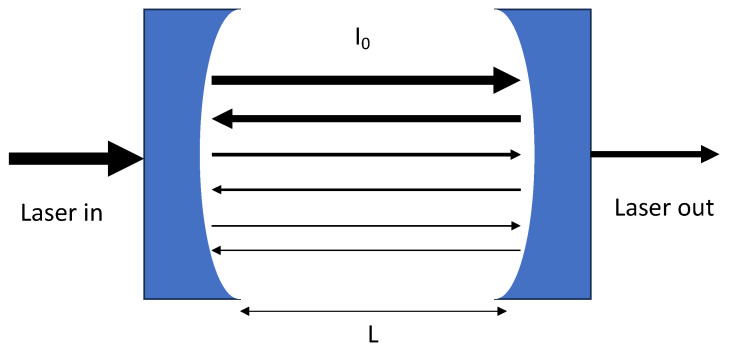
Schematic of light in a high-finesse (typically *R* of >99.99%) optical cavity showing multiple passes of light within the cavity.

CRDS is a powerful spectroscopic technique notable for its remarkable sensitivity and versatile application areas [20,21]. Devised initially to measure dielectric mirror reflectivity, CRDS utilizes a stable optical resonator constructed from highly reflective mirrors [22], typically with reflectivity (*R*) of ~99.99%, as shown in Figure 3. Typically, the high reflectivity is due to specialized multi-layer optical coatings on the inner (cavity-facing) mirror surfaces. The sample (causing absorption and/or scattering) is housed within the high-finesse optical cavity. A beam is injected into the cavity and undergoes multiple reflections between the mirrors, where its intensity gradually decays due to the combination of mirror loss and absorption and/or scattering induced by the sample (i.e., air containing PM and/or AMCs in our case). Sample quantification is based on measuring the decay times of the light in the cavity based on the Beer–Lambert law, as is detailed below. The high sensitivity of CRDS is derived from its long effective path length (due to the many light passes within the optical cavity), enabling the detection of very low sample densities [20,21].

The Beer–Lambert law provides a relationship between a substance’s concentration and the light it absorbs [20]. For cases involving both absorption and scattering (as is the case with particles), a total extinction coefficient per unit length *k* can be used:

(1) $$k=k_{abs}+k_{scat}$$

where $k_{abs}$ represents the absorption coefficient and $k_{scat}$ represents the (Mie) scattering coefficient. By measuring the total extinction coefficient (k), and knowing the spectroscopic parameters (species absorption or scattering cross-section), it is possible to determine the density and concentration of target analyte species. The temporal decay of light intensity in the cavity follows an exponential behavior over time, characterized by the 1/*e* time, which is referred to as the ring-down time, *τ*. In the absence of any absorbing (or scattering) species, the decay time is referred to as the empty-cavity ring-down time, $\tau_{0}$. In the presence of an absorbing (or scattering) sample, *τ* is shortened due to additional optical loss. The extinction coefficient, k, of the sample, is found from the change in decay time caused by the sample interaction:

(2) $$k=\frac{\left(1-R\right)}{L}\left(\frac{\tau_{0}-\tau }{\tau }\right)$$

where *L* is the length of the cavity (and it is assumed that the sample is uniformly present over the length of the cavity).

Past work from several groups, including ours, has shown CRDS detection of HCl as applied to atmospheric research. Hagen et al. showed closed-path CRDS measurements of HCl (in ambient air) with a detection limit of ~20 parts per trillion (ppt) over a duration of 1 min [23]. However, the closed-path system used by Hagen at al. does not directly translate to the present mobile cleanroom application, as it requires a relatively bulky and power-intensive flow system (vacuum pump). In other work, Panu et al. utilized a CRDS analyzer based on a diode laser operating at 1742 nm and a bismuth-doped fiber amplifier to detect HCl in cleanroom environments, achieving a detection limit of ~3 ppb (for 1 min integration time) [24]. In contrast to past efforts, the present contribution aims to develop a compact sensor, suitable for cleanroom implementation, that can, for the first time, simultaneously detect both AMCs and PM with a single instrument.

While the vast majority of CRDS development has been for gas-phase detection, there have been limited studies of PM detection due to optical extinction from Mie scattering [25,26,27,28,29,30,31]. These contributions do not directly provide an appropriate system for cleanroom monitoring (amenable also to AMC detection) but do offer a point of departure for our efforts. The contributions from Smith et al., Pettersson et al., and Gordon et al. both seek to determine the overall Mie scattering loss of air samples due to particles with the studies oriented to atmospheric chemistry and effects on climate change [25,26,27]. Other studies have attempted to retrieve optical properties of aerosols such as the extinction coefficient, extinction cross-section, extinction efficiency and refractive index [29,30,31]. Past work from McHale et al. has shown that Mie scattering induced reduction and fluctuation in ensembles of ring-down times due to airborne particles [32]. The ring-down fluctuations were considered a noise source that reduced the sensitivity of gas-phase detection, while in the present work, we seek to analyze the ring-down ensembles (fluctuations) to enable the quantification of particle densities in cleanroom air samples.

## 3. Experimental Methods

### 3.1. AMC and PM Testbed Chamber

**Figure 4:**
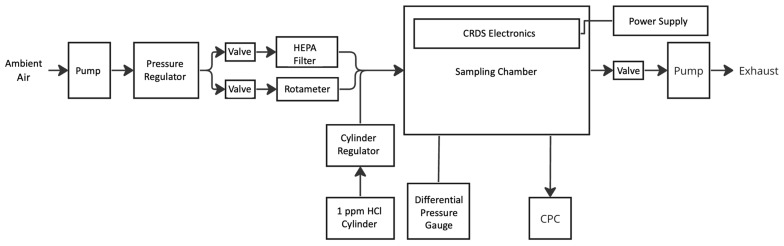
Schematic of the delivery system and control chamber.

A testbed chamber, shown in Figure 4, was developed to achieve controlled HCl (as a representative AMC) and PM concentrations. The main chamber is a 90 cm × 60 cm × 30 cm airtight acrylic box. Ambient lab air is pulled with a pump (Gast 86R123-101-N170X, Arvada, CO, USA) through a pressure regulator (Fairchild model 30232, Winston-Salem, NC, USA) to maintain a steady airflow of 22 L/min. This primary flow is divided into two lines with flow rates that are individually controlled by needle valves. The first line passes through an HEPA filter (PALL 12144, Port Washington, NY, USA), which removes >99.99% of particles equal to or greater than 0.3 μm in diameter. The second line transmits (unfiltered) ambient sample air with an additional rotameter (Dwyer RMA-151-SSV, Michigan City, IN, USA) for precise flow rate control. These two lines are then joined again before passing into the control chamber. By changing the ratio of the two flows (with control valves and a rotameter), varying PM number concentrations (ISO levels for PM) can be delivered to the main chamber. A condensation particle counter (CPC—TSI Condensation Particle Counter 3787, Shoreview, MN, USA) connected to the chamber is used as a particle concentration reference measurement.

For HCl (AMC) testing, a third line attached to a reference gas HCl cylinder (typically 1 ppm HCl in balance air) is employed. A cylinder regulator (Harris, CGA 330. Mason, OH, USA) controls the delivered HCl flow rates, allowing controlled dilution in combination with the other two lines (e.g., by a factor of 100 to achieve an HCl concentration of 10 ppb). The inlet and outlet flows to the chamber need to be balanced to maintain steady-state conditions at the appropriate chamber pressure, which is monitored with a differential pressure gauge (Magnehelic 2000-00N, Michigan City, IN, USA). The chamber outlet flow is regulated with a control valve connected to a pump (Varian IDP2B01, Santa Clara, CA, USA) that extracts air from the chamber (to an exhaust). The time required for the chamber to respond (equilibrate) to changes in HCl concentration is approximately 10 min. Particle tests are performed at chamber pressures very close to ambient pressure (84 ± 1 kPa in Fort Collins, CO, USA). All the tests were conducted at ambient laboratory temperature of 24 ± 1 °C. For HCl tests, the chamber is run at a very slight vacuum (i.e., a vacuum pressure of ~10 Pa) to reduce the risk of fugitive HCl escaping the enclosure and entering the lab. The chamber also houses the open-path CRDS optical head and optoelectronics with external cabling (for power, etc.) that runs through the chamber wall via a sealed feedthrough.

When chamber operation is first initiated, the chamber initially contains unfiltered ambient lab air (with high and uncontrolled particle levels at ISO >~10). After approximately 45 min of operation using the flow configuration described above, the chamber can attain an ISO level of ~5. It takes another 45 min to bring the chamber to more pristine particle levels of ISO ~3. Note that measuring the PM levels with the CPC at these relatively low particle densities (ISO levels) is challenging, such that sequential sampling (with longer integration times) was utilized following an approach derived from ISO 14644-1 (Annex D) [3].

### 3.2. Open-Path CRDS Instrument for HCl and PM Detection

**Figure 5:**
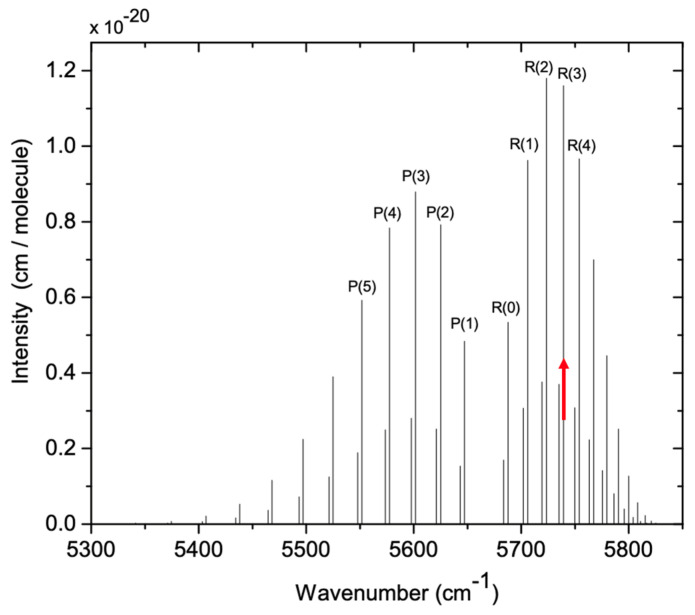
Line strengths of rotational lines of the 2-0 vibrational absorption band of H^35^Cl and H^37^Cl. The CRDS sensor uses the R(3) line of H^35^Cl at 5739.26 cm^−1^ (indicated with red arrow) [23].

The CRDS setup presented here derives from our previous work [23,33] but with modifications and advances for the cleanroom application. The instrument targets the R(3) H^35^Cl line within the (2-0) absorption band [34] due to its relatively strong line strength, separation from other atmospheric absorbers, and compatibility with widely used near-infrared (NIR) optoelectronic components. The (2-0) band corresponds to molecular transitions from the initial vibrational level $v^{\prime \prime }$ = 0 (vibrational ground state) to the final level $v^{\prime }$ = 2 as a photon is absorbed. Figure 5 provides a stick-plot of the line strengths of the rotational lines of the band with the first 5 P- and R-branch transitions (absorption lines) labeled. Each transition consists of two closely situated lines, representing absorption by the two isotopologues H^35^Cl and H^37^Cl. The measured concentrations account for isotopic abundances (0.758 for H^35^Cl and 0.242 for H^37^Cl), providing information about the overall HCl population, encompassing both isotopologues. This specific absorption line has also been adopted by other researchers for HCl absorption measurements [24,35].

**Figure 6:**
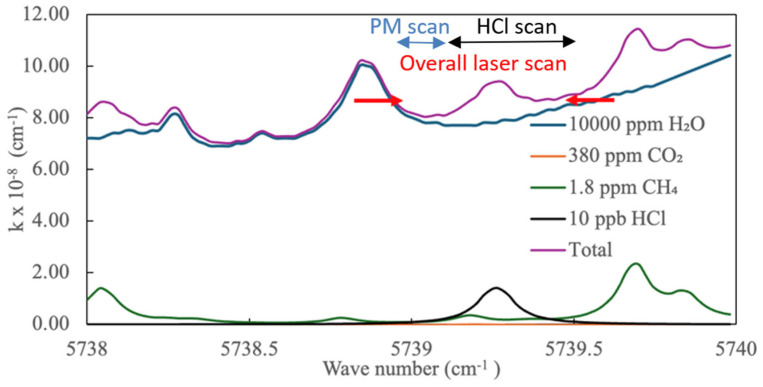
Simulated absorption spectra with 10 ppb HCl, 1% H_2_O (~50% relative humidity), 1.8 ppm CH_4_, and 380 ppm CO_2_ at pressure of 1 atm and temperature of 296 K. The red arrows highlight the scan region.

It is crucial to achieve separation from the absorption lines of other potential interfering molecules to ensure the success of optical measurements. We have considered potential interferences from potential ambient molecules and their corresponding isotopologues, including methane (CH_4_), carbon monoxide (CO), carbon dioxide (CO_2_), water (H_2_O), nitrogen monoxide (NO), and dinitrogen oxide (N_2_O) at typical atmospheric concentrations. Figure 6 shows HCl simulation along with other molecules having nearby absorption features. Voigt lineshapes are used with ambient pressure and temperature. Importantly, water vapor does lead to an overall increase in absorption at the location of the HCl peaks, but the water baseline (even for 1% water vapor) is relatively flat and does not significantly affect the HCl peak fitting.

Following typical methods of diode laser spectroscopy, we set the diode temperature to coarsely center the overall wavelength region combined with the (sawtooth) current modulation to precisely scan the laser wavelength. Different experimental objectives can employ different laser scan parameters, but a typical scan region spans from ~5738.95 to 5739.5 cm^−1^ (corresponding to current range of ~80–91 mA for our specific laser) to allow both PM and HCl detection, as shown in Figure 6. The PM detection exploits the spectrally flat region of the spectrum from ~5738.95 to 5739.1 cm^−1^ to provide a stable baseline against which the (Mie scattering induced) changes to ring-down times can be examined. (Detailed examination shows that the spectrum here is not exactly flat, which is mostly due to water absorption; however, simulation shows that even for relative humidity of ~50%, the baseline absorption ripple is <2 × 10^−9^ cm^−1^ which, when converted to ring-down time variation for our cavity, corresponds to a range of <0.6 μs, which is negligible relative to the observed ring-down time distributions.) HCl detection is based on recording the profile of the HCl absorption feature based on the remaining portion of the laser scan from ~5739.1 to 5739.5 cm^−1^. A LabVIEW MathScript (National Instruments) code is used for spectral fitting to infer the HCl concentration via eqn. (2). A typical laser scan rate is 5 s^−1^, which corresponds to ~20 ring-downs per second for our conditions. In the present work, owing to our inability to simultaneously control both PM and HCl levels (since our HCl cylinder uses particle-laden air which is difficult to filter due to adsorption (“sticky-gas”) effects of HCl), we scan the laser over the aforementioned range but separately perform the HCl and PM experiments; however, simulations show the scheme should be robust for simultaneous detection also. Validating HCl measurements (at known densities) were performed following our past methods [23].

**Figure 7:**
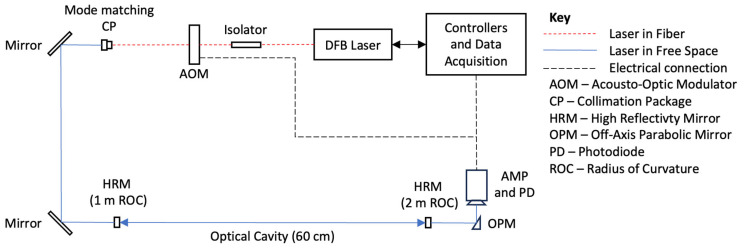
Optical components and data acquisition system of the HCl sensor.

Figure 7 shows the schematic of the optical components and data acquisition system used for our CRDS setup. We utilize a distributed-feedback diode laser in a 14-pin butterfly package (KELD1F5DAAA, NEL Lasers, Yokohama, Japan) with a center wavelength of ~1742 nm, linewidth of ~2 MHz (negligible spectral broadening by the laser), and output power of ~15 mW from a single-mode fiber pigtail. An integrated thermoelectric cooler (TEC) affixed to the diode regulates the laser temperature, ensuring wavelength stability. Current scanning is used to wavelength scan the laser (parameters given above). An acousto-optic modulator (AOM—Brimrose, 1801-SY-16708, Baltimore, MD, USA) is used as an optical switch to extinguish light entering the cavity, allowing recording of the optical ring-down decay. The AOM is triggered via the data acquisition (DAQ) system, as the laser overlaps with the cavity resonances, causing a rapid extinction with time constant of ~300 ns [23]. Following the AOM, the laser beam passes through an aspheric collimation lens (Thorlabs, CFC-11X-C, focal length = 11 mm, Newton, NJ, USA) for mode-matching to the cavity. Precisely positioning this lens relative to the fiber output is essential for achieving spatial mode matching, i.e., overlapping and aligning the beam spatially with the TEM_0,0_ mode of the cavity [36]. Two (intermediately located) steering mirrors are used for delivery and alignment to the optical cavity. The optical cavity itself consists of two high-reflectivity (HR) dielectric mirrors (Advanced Thin Films, Longmont, CO, USA), separated by ~60 cm, with the input mirror having a curvature of 1 m and the output mirror having a curvature of 2 m. (These mirror radii allowed shorter path lengths, to achieve mode match, relative to other available choices.) Experimental measurements yield mirror reflectivity of R = 99.9981% due to the empty-cavity ring-down time, t_0_, of 105 μs, which is close to the manufacturer’s specifications. To detect the weak NIR light exiting the cavity, an extended wavelength InGaAs photodiode (G8421-03, Hamamatsu, Bridgewater, NJ, USA) is employed, which is connected to a transimpedance amplifier (341-4-inv-10PF, Analog Modules, Longwood, FL, USA). This amplifier has gain adjusted to 2 × 10^6^ V/A and a bandwidth of 2 MHz, ensuring that the ring-down signals are accurately captured with minimal distortion [23].

A core electronics platform is established through the integration of a tailored carrier board with an sbRIO-9651 (National Instruments, Austin, TX, USA) system, which is based on the Xilinx Zynq-7020 (AMD, Santa Clara, CA, USA) system on chip. This configuration served the purpose of detecting and processing analog signals originating from the photodetector. At mode match conditions, these signals would peak at about 1 V. A LabVIEW (LabVIEW 2019, Austin, TX, USA) program (developed by TCB Engineering) is employed to manage the instrument, including AOM triggering, ring-down signal acquisition, and data acquisition and processing. For ring-down data collection for particles (as opposed to HCl), a slightly different setup was used. In this case, the cavity detector voltage signal was digitized by a 2.5 MS s^−1^, 14-bit PCI multi-purpose DAQ card (PCI-6132, National Instruments, Austin, TX, USA), which also controlled the AOM.

## 4. Results and Discussion

### 4.1. Stability of HCl Detection

**Figure 8:**
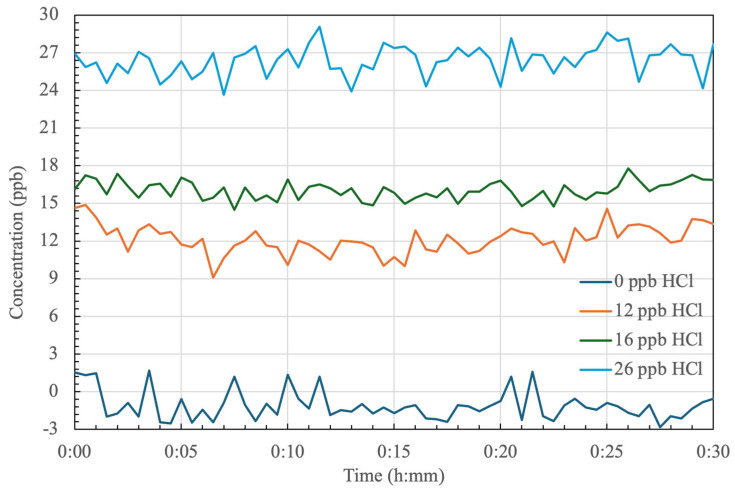
Time-series of HCl concentration measurements by CRDS for several fixed concentrations (shown in legend).

We have performed a series of open-path CRDS tests to measure HCl spectra at different delivered concentrations. Figure 8 shows examples of time-series (30 min) of HCl concentrations recorded at low ppb ranges. The tests were conducted with the detailed flow dilution and optical setups described in Section 3.1 and Section 3.2, respectively. Each test was a distinct 30 min trial with tests performed over several days at ambient pressure of 84 ± 1 kPa and temperature of 24 ± 1 °C. The standard deviation of each test run was ~1.2 ppb HCl. Note that our controlled dilution scheme allowed rough, but not precise, setting of the delivered HCl concentration such that the reported concentrations for the curves in Figure 8 are due to the CRDS measurements themselves, which is justified by CRDS being a self-referencing technique. The accuracy of HCl determination with the given transition has been extensively validated with calibration gases in our past work (with a similar closed-path CRDS system) showing measurement accuracy of better than 10% [23].

To determine the optimal integration (averaging) time for the CRDS system, we conducted an Allan Variance analysis on a series of concentration measurements using a customized approach outlined in Huang and Lehmann [37]. This modified method produced a significantly more refined Allan Variance curve compared to the conventional two-sample variance approach. The capacity of a CRDS sensor to ascertain concentrations of trace atmospheric elements hinges on the optical precision of the instrument and the spectroscopic correlation between optical absorption and element concentration. While extended integration times typically yield enhanced detection thresholds, system drift eventually begins to dominate, leading to an optimal, finite integration time.

**Figure 9:**
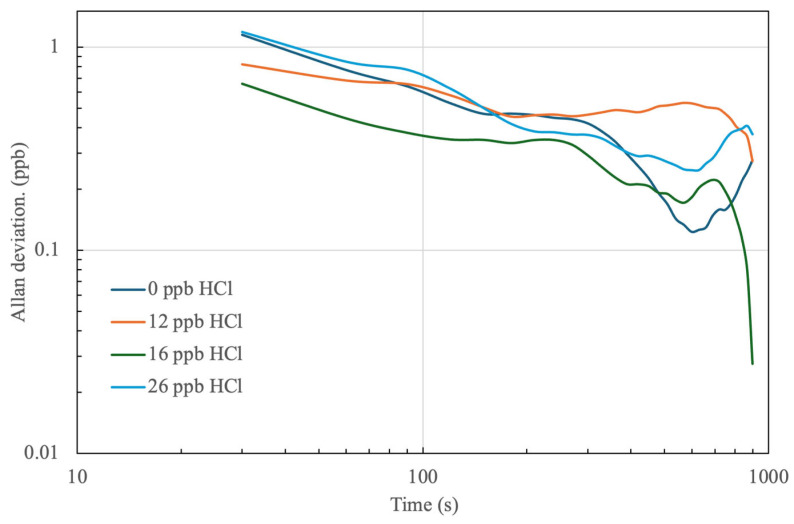
Allan variance of HCl concentration due to measurements at 4 concentration levels.

The Allan variance of the four tests from Figure 8 is shown in Figure 9. For all four cases studied, the Allan variance for an integration time of 30 s is ~1 ppb. For the 0 ppb case, the Allan variance exhibits a minimum of around 0.15 ppb at an optimal integration time of roughly 10 min, while the Allan variance results for the other concentrations show similar results (i.e., all have variance of <1 ppb at 10 min). These values demonstrate favorable performance at practical time scales relative to the typical cleanroom monitoring requirements for HCl levels. Because our sampling chamber needs ~10 min to equilibrate to HCl concentration changes, we cannot directly measure the sensor response time in this configuration; however, open-path CRDS with similar systems (e.g., [20,33]) has allowed sampling at ~1 Hz (with a true time-response of ~1 s) as can be expected given the lack of flow cells. Practically speaking, longer measurement times will be required depending upon the needed signal-to-noise levels.

### 4.2. Particle Detection

Our approaches for PM detection with open-path CRDS are based on the statistical examination of ensembles of individual ring-down times (where the increased/varying optical extinction due to Mie scattering perturbs the ring-down times). As described in Section 3, reference (truth) measurements are made by a CPC connected to the chamber. We recorded multiple time-series of ring-down times and particle number concentrations (ISO level) simultaneously. From these data streams, we extracted ensembles of 15,000 ring-downs (at constant ISO levels), each of which corresponds to ~15 min of data collection.

We report on the use of centralized standard moments, of various orders, to extract information on the shapes of the ring-down time distributions (which carry information on the particles due to the Mie scattering effects). For a distribution *P*(*x*), the (unnormalized) standard moment, *μ_n_*, of degree *n* is found as

(3) $$\mu_{n}=\int_{-\infty }^{\infty }{\left(x-\mu \right)}^{n}P\left(x\right)dx$$

where *μ* is the mean (non-centralized first-moment). The centralized moments, of differing orders, carry different information. The centralized first-moment is, by definition, zero. The second central moment, also referred to as the variance, reflects the spread (width) of the distribution. The third central moment provides a measure of the skewness or asymmetry to the distribution. Finally, the fourth central moment provides a measure of the kurtosis, or “heavy-tail”, of the distribution. We hypothesize that the second central moment may increase with ISO (since there will be more spread in ring-down times when there are increasing counts due to signals with Mie scattering contributions). A similar logic leads to the hypothesis that the 4th moment may increase with ISO. Likewise, the 3rd-order moments have negative amplitudes but with higher values hypothesized for the increased ISO (where the distribution is expected to have a longer tail on the high ring-down side).

**Figure 10:**
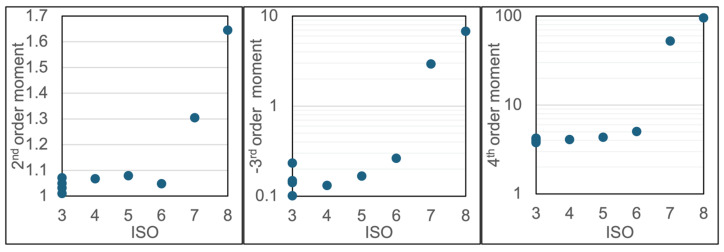
Higher-order central moments for ensembles of ring-down time data for varying particle levels in the range of ISO 3–8.

**Figure 11:**
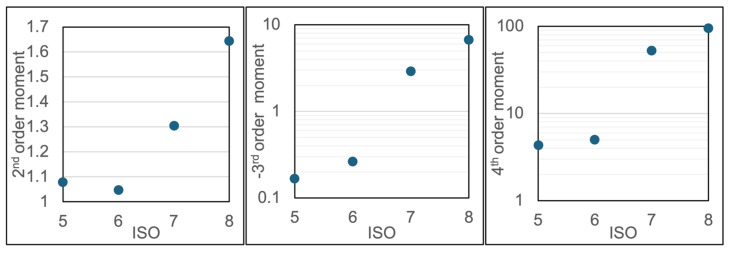
Higher-order central moments for ensembles of ring-down time data for varying particle levels in the range of ISO 5–8. Clear trends in the moments versus ISO are apparent. Correlation coefficients are 0.907, (−)0.936, and 0.948 for the 2nd/3rd/4th order central moments versus ISO.

Figure 10 shows plots of the 2nd, 3rd, and 4th-order centralized moments for ensembles of ring-down times collected over the range of ISO from 3 to 8. These data show a weak (or negligible) dependence of the higher-order moments in the range of ISO ~3–5 but then clear trends with increasing moments at higher ISO levels in the ISO ~5–8 range. These results are promising in terms of using the ring-down times to infer the particle ISO levels. It is anticipated that with future sensitivity improvements, i.e., the ability to measure ring-down times with more precision and greater baseline stability, we can also establish similar trends at lower ISO levels. Figure 11 is another data set focusing on ISO 5 to ISO 8 and showing clear trends of the higher-order moments versus ISO level. Correlation coefficients are provided in the figure caption and support the validity of the hypotheses (over this ISO range).

Similar to the HCl testing, there was some variability in the particle concentrations achieved in each PM experiment. However, the single particle detection approach used by the CPC combined with extended sampling times allows for high accuracy in concentration estimates and a better representation of particle concentrations over time. The chamber was operated at concentrations far below the upper limit of the CPC, minimizing concerns about sampling bias due to coincidence errors. The testing presented here aimed to determine the operational range of the CRDS, focusing on concentrations significantly different from the ISO tier transition points. Future testing will systematically target concentrations near each ISO tier rating threshold (e.g., the transition between ISO 5 and ISO 6) to quantify CRDS bias and error. This approach will address the potential misidentification of tiers and determine the sampling duration necessary to achieve reliable measurements with sufficient counting statistics.

## 5. Conclusions and Future Work

We have demonstrated the possibility of using a relatively simple open-path cavity ring-down spectroscopy (CRDS) instrument to make simultaneous measurements of both PM and trace levels HCl gas, where HCl is relevant as a representative atmospheric molecular contaminant (AMC). Using a single compact instrument for this type of cleanroom monitoring can be enabling in terms of modern, advanced cleanrooms using mobile (gantry-based) sensing, which allows a more efficient deployment monitoring of multiple, distributed assets (equipment groups).

The system presented here leverages widely available (and relatively inexpensive) NIR components, which were extensively developed by the telecom industry. The performance metrics of our instrument should satisfy cleanroom requirements and be competitive relative to other monitoring devices. For AMC detection, we can achieve an Allan variance (limit of detection) for HCl of around 1 ppb (in 30 s measurement times), which should be very adequate for measurements in cleanroom conditions. The lowest possible detection limits occur for integration times of approximately 10 min and are <1 ppb. The needed detection limits for cleanroom monitoring vary with AMC species and product type with detection limits of ~1 ppb, as reported here, being useful for many practical applications.

We have investigated and presented novel approaches toward inferring particle density (and associated ISO PM level) from ensembles of ring-down times based on signal influences (increased optical extinction) due to the Mie scattering of the CRDS probe beam. In particular, we look at higher-order (2nd–4th) central moments of the ring-down time distributions, as these moments carry information on the width, kurtosis and tail of the distribution. Our results show that the higher-order moments clearly respond to particles, following the provided hypotheses, even at a very low particle density of ~10^5^ m^−3^ (ISO 5), and with clear dependences at yet higher levels. The detection of particles at these low levels is useful in numerous cleanroom applications, especially in the subfab region.

When transitioning from a validation phase to a field-deployable solution, concessions to ideal sampling conditions are often necessary due to the practical challenges encountered in real-world environments. These compromises can affect the accuracy and precision of sampling, making it essential to understand the limits and capabilities of the deployed system. A critical component of future work will involve rigorously evaluating the sampling accuracy and precision achievable in field conditions to ensure reliable and consistent data collection.

Future work will examine performance improvements of the CRDS instrument with goals of great practicality of operation (for example, over larger temperature windows) along with increased sensitivity to detect PM and AMCs at lower levels. For example, the integration of an external optical isolator, in addition to the isolator that is built into the laser, can improve the system sensitivity (by reducing feedback from the cavity to the laser) [38]. Via modifications to the HCl/PM sampling chamber, we will also explicitly show simultaneous HCl/PM detection using the same laser scan scheme presented here. Overall, the possibility of sensitive and compact instrumentation for AMC and particle detection has the potential to be enabling for next-generation mobile monitoring systems (Figure 2), offering greatly increased spatial coverage (field of view) relative to traditional stationary monitoring. An upcoming step will be to trial the open-path CRDS instrument in actual cleanroom field settings.

## Data Availability

Data are available upon request to the author.
